# Chlorogenic acid attenuates pyrrolizidine alkaloid-induced liver injury through modulation of the SIRT1/FXR signaling pathway

**DOI:** 10.1186/s13020-025-01077-2

**Published:** 2025-03-12

**Authors:** Jie Xu, Qiongwen Xue, Aizhen Xiong, Yilin Chen, Xunjiang Wang, Xing Yan, Deqing Ruan, Yumeng Zhang, Zhengtao Wang, Lili Ding, Li Yang

**Affiliations:** 1 Shanghai Key Laboratory of Complex Prescriptions, The MOE Key Laboratory for Standardization of Chinese Medicines and the SATCM Key Laboratory for New Resources and Quality Evaluation of Chinese Medicines, Institute of Chinese Materia Medica, Shanghai University of Traditional Chinese Medicine, Shanghai, 201203 China; 2 Department of Pharmacy, Seventh People’s Hospital of Shanghai University of Traditional Chinese Medicine, Shanghai, 200137 China; 3 State Key Laboratory of Discovery and Utilization of Functional Components in Traditional Chinese Medicine, Shanghai University of Traditional Chinese Medicine, Shanghai, 201203 China

## Abstract

**Background:**

Pyrrolizidine alkaloids (PAs), recognized globally for their hepatotoxic properties, significantly contribute to liver damage through an imbalance in bile acid homeostasis. Addressing this imbalance is crucial for therapeutic interventions in PA-related liver injuries. Chlorogenic acid (Cga), a phenolic compound derived from medicinal plants, has demonstrated hepato-protective effects across a spectrum of liver disorders. The specific influence and underlying mechanisms by which Cga mitigates PA-induced liver damage have not been clearly defined.

**Materials and methods:**

To explore the protective effects of Cga against acute PA toxicity, a murine model was established. The influence of Cga on bile acid metabolism was confirmed through a variety of molecular biology techniques. These included RNA sequencing, western blotting, and immunoprecipitation, along with quantitative analyses of bile acid concentrations.

**Results:**

Our findings indicate that Cga enhances sirtuin 1 (SIRT1) activation and increases farnesoid X receptor (FXR) signaling, which are crucial for maintaining bile acid balance in PA-induced hepatic injury. When mice subjected to PA-induced hepatic injury were treated with SIRT1 inhibitors alongside Cga, the hepatoprotective effects of Cga were significantly reduced. In *Fxr*-KO mice, the ability of Cga to mitigate liver damage induced by PAs was substantially reduced, which underscores the role of the SIRT1/FXR signaling axis in mediating the protective effects of Cga.

**Conclusion:**

Our research suggests that Cga can serve as an effective treatment for PA-mediated hepatotoxicity. It appears that influencing the SIRT1/FXR signaling pathway might provide an innovative pharmacological approach to address liver damage caused by PAs.

**Supplementary Information:**

The online version contains supplementary material available at 10.1186/s13020-025-01077-2.

## Introduction

Pyrrolizidine alkaloids (PAs) are alkaloids that typically contain either a saturated or a 1,2-unsaturated necine base unit and are derived primarily from plants across *Asteraceae* (*Compositae*), *Boraginaceae,* and *Fabaceae* (*Leguminosae*) [1]. These compounds, with more than 500 distinct PA N-oxides identified and PAs, are estimated to exist in more than 6000 plant species on the basis of chemotaxonomic data [2]. The ingestion of PAs can lead to severe hepatotoxic effects. Notably, acute exposure to PAs frequently results in significant liver damage, characterized by hepatic sinusoidal obstruction syndrome (HSOS). This condition often presents with symptoms such as hepatomegaly, ascites, and hyperbilirubinemia [3, 4]. Research has established that exposure to PAs is a primary cause of HSOS [4, 5]. More than 15,000 incidents of PA poisoning have been documented worldwide, with reports from countries such as China, Germany, Britain, Jamaica, South Africa, India, Switzerland and the United States [6]. In China, the predominant source of HSOS is linked to the consumption of *Gynura japonica* (Thunb.) Juel (*G. japonica*), which is involved in 50–89% of all HSOS incidents [7]. According to our previous research,* G*. *japonica* contributes to liver damage through both direct hepatocyte injury and the induction of cholestasis which notably disrupts bile acid homeostasis [8]. Thus, PAs pose significant risks as hepatotoxins, highlighting the urgent need for the development of new preventive and therapeutic strategies to address the hepatic damage they induce.

Bile acids, which are significant metabolites of endogenous cholesterol, maintain a balanced state in the hepatic and peripheral circulation through their existence as both conjugated and free forms, such as glycine conjugates and taurine conjugates. Previously, we demonstrated that after PA exposure, the detoxification process involving the conjugation of free bile acids to taurine and glycine via bile acid-CoA amino acid N-acetyltransferase was hindered. This interference results in increased concentrations of bile salts within hepatocytes, which exacerbates liver damage [9]. Bile acid homeostasis is dependent on specific transport mechanisms in enterocytes and hepatocytes. Conjugated bile acids are excreted into the bile duct by the canalicular membrane transporter bile salt export pump (BSEP) along with multidrug resistance-associated protein 2 (MRP2) in hepatocytes, which are essential for bile flow regulation [10]. Most bile acids are predominantly reabsorbed in the ileum by the apical sodium-dependent bile acid transporter (ASBT). The organic solute transporter α and β heterodimer (OSTα/β) in the basolateral membranes of enterocytes induces bile acid efflux into the portal circulation. These transporters, while secondary under normal physiological conditions, can become critical under cholestatic states [11, 12]. The farnesoid X receptor (FXR) orchestrates gene transcription for these transporters, which is crucial for maintaining bile acid metabolic equilibrium. As an orphan nuclear receptor, FXR significantly influences bile acid homeostasis by transcriptionally regulating bile acid synthesis, influx, efflux, and detoxification processes in the enterohepatic pathway [13]. The regulation of FXR activity involves a dynamic acetylation/deacetylation process mediated by p300 and Sirtuin 1 (SIRT1) [14]. SIRT1, a nicotinamide adenine dinucleotide^+^-dependent histone III deacetylase with evolutionary conservation, becomes activated under energy-deprivation conditions, influencing pivotal metabolic functions, including the metabolism of bile acids [15, 16]. Hepatic SIRT1 deficiency leads to increased bile acid concentrations and decreased activity of FXR and its target genes, such as small heterodimer partner (SHP) [14, 17]. A lack of intestinal SIRT1 expression leads to decreased hepatic bile acid levels as well as decreased expression of ileal bile acid metabolic genes such as OSTα and ASBT [18]. Furthermore, SIRT1 deacetylates FXR, increasing its ability for gene transcription and DNA binding [14]. Therefore, targeting the FXR/SIRT1 pathway may offer therapeutic potential for addressing disturbances in bile acid homeostasis induced by PAs.

Chlorogenic acid (Cga), a phenolic constituent, is prevalent in several medicinal botanicals, such as *Lonicera japonica* Thunb. and *Eucommia ulmoides* Oliv. This compound also appears extensively in various fruits, vegetables, and popular beverages, such as coffee [19]. Recent studies have established that Cga has a range of pharmacological effects. These include the ability to mitigate inflammation, reduce fever, scavenge free radicals, manage lipid levels, and exhibit properties that are antioxidative, antiviral, and antihypertensive [20]. Previous studies have demonstrated the hepatoprotective ability of Cga in mitigating liver damage caused by a range of hepatotoxicants, including monocrotaline-induced HSOS, acetaminophen-induced liver toxicity, and liver injuries triggered by endotoxins [21–23]. Hence, we hypothesized that Cga has potential as an effective agent for mitigating liver damage caused by PAs.

This investigation focused on the role of Cga in managing bile acid equilibrium during PA-induced hepatic damage while elucidating the mechanisms involved. These findings underscore the importance of triggering the FXR/SIRT1 pathway, which is critical for maintaining hepatic functionality under the stress of PA-induced hepatotoxicity.

## Materials and methods

### Reagents

*G. japonica* total alkaloid extract (TA) was acquired from Chengdu Biopurify Phytochemicals Ltd. (Chengdu, China). To confirm the adequate extraction of active components, the PA content within the TA was evaluated using a previously described method [8]. First, the TA was solubilized in 5% HCl, and the pH of the solution was meticulously adjusted to a neutral range of 6–7 by the addition of 5 mM NaOH. Finally, to achieve the desired working concentration, saline was incorporated into the TA mixture. The Cga purified for this investigation was obtained from Shanghai Standard Technology Co. Ltd. (Shanghai, China).

### Animal experiments

C57BL/6J male mice aged eight weeks were procured from the Laboratory Animal Center at the Shanghai University of Traditional Chinese Medicine (SHUTCM). The experiment was conducted with mice housed under conditions free from specific pathogens. These conditions included a stable temperature maintained at 23 ± 2 ℃ and a consistent relative humidity of 55 ± 5%. The ventilation in the room was adjusted to cycles between 12 and 18 times per hour, complemented by a light/dark cycle maintained for 12 h each. The mice were maintained on a standardized diet provided in the laboratory, with free access to tap water. Oversight by the SHUTCM Experimental Animal Ethical Committee ensured that humane care was uniformly administered to all the subjects.

According to our research group’s previous studies, a mouse model of acute liver injury was established using TA [24]. Initially, the study involved dividing thirty mice into five distinct groups, each consisting of six individuals. These groups included vehicle, TA, and different concentrations of Cga (20 mg/kg, 40 mg/kg, and 80 mg/kg). Treatment commenced with a single dose of 100 mg/kg TA or the corresponding vehicle, which was administered intragastrically. Subsequent doses of Cga were administered at 6 h and again at 30 h following the initial TA administration. At 48 h post-TA administration, the animals were euthanized for immediate collection of samples from the blood, liver, ileum, and feces.

In a subsequent phase of the experiments, wild-type (WT) mice and whole-body *Fxr* knockout (*Fxr*-KO) mice were allocated into six distinct clusters, each containing six mice. These groups were designated WT-vehicle, WT-TA, WT-TA + Cga, *Fxr*-KO-vehicle, *Fxr*-KO-TA, and *Fxr*-KO-TA + Cga. A single dose of 100 mg/kg TA or the corresponding vehicle was administered intragastrically, followed by two subsequent doses of Cga (40 mg/kg each), which were administered at 6 h and 30 h after the initial TA dose. After 48 h of TA administration, all the mice were sacrificed, and both blood and liver samples were subsequently collected for subsequent analysis.

In the third experimental series, a total of 30 mice were distributed into five separate groups, with each group comprising six animals: (1) vehicle, (2) TA, (3) TA + Cga, (4) TA + Cga + EX 527, and (5) EX 527 (HY-15452, MedChemExpress, Shanghai, China) groups. A single oral administration of 100 mg/kg TA or its corresponding vehicle was given. Two doses of Cga (40 mg/kg) were orally administered at 6 and 30 h following TA treatment. EX 527 was administered via intraperitoneal injection at a dose of 20 mg/kg one hour before the initial Cga treatment commenced. Two days after the TA was applied, the mice were sacrificed to facilitate the immediate collection of both liver and blood samples.

### Serum biochemistry analysis

To determine the levels of alanine aminotransferase (ALT), aspartate aminotransferase (AST), and total bile acid (TBA) in the serum, fresh blood samples were processed to isolate the serum. An Enzymatic Assay Kit from Nanjing Jiancheng Bioengineering Institute (Nanjing, China) was subsequently used to measure the concentrations of ALT, AST, and TBA in strict accordance with the instructions provided by the manufacturer.

### Histopathological analysis

Freshly obtained liver samples were preserved in 4% paraformaldehyde before being encapsulated in paraffin. Staining of the paraffin sections was performed using hematoxylin and eosin (H&E), which enabled detailed observation through a light microscope (Olympus Corp., Japan).

### Quantitative real-time PCR (qRT-PCR) analysis

Hepatic samples were processed to extract RNA via an EZB-RN001-plus kit supplied by EZBioscience in the USA. The subsequent synthesis of cDNA was facilitated by the addition of Evo M-MLV RT Premix (AG11706, Accurate Biology, Hunan, China), which adheres to the protocols recommended by the manufacturer. The quantitative analysis of mRNA was conducted with a SYBR^®^ Green qPCR Kit (AG11718, Accurate Biology, Hunan, China) with a real-time PCR detector. The expression levels of specific genes were normalized to those of GAPDH via the 2^**−ΔΔCt**^ method and are reported as a ratio to the gene expression level of the vehicle group. The primer sequences are provided in Table 1.

**Table 1:**
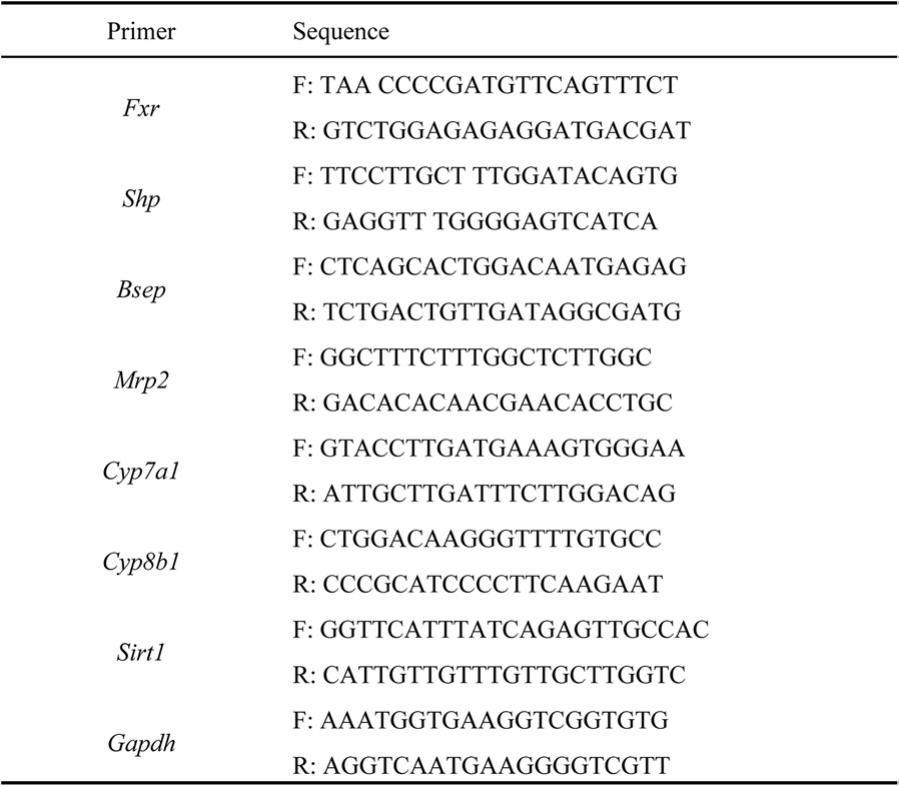
Primer sequences for qRT-PCR

### RNA-sequencing analysis

RNA was extracted from freshly obtained liver samples using TRIzol reagent according to the manufacturer’s instructions. The total amount and integrity of the extracted RNA were determined using an RNA Nano 6000 Assay Kit with a Bioanalyzer 2100 system (Agilent Technologies, USA). This RNA served as the input material for subsequent sample preparation steps. The mRNA purification procedure included several steps: the use of poly-T oligo-attached magnetic beads, followed by fragmentation, reverse transcription, end repair, the addition of a single ‘A’ base, adaptor ligation, purification, and enrichment via PCR. The resulting library fragments were then purified using the AMPure XP system (Beckman Coulter, USA), and the final library quality was assessed using an Agilent 2100 Bioanalyzer. High-quality libraries were sequenced on an Illumina NovaSeq 6000 platform.

### Western blotting (WB)

Liver tissue samples were homogenized using RIPA buffer (89900, Thermo Fisher Scientific, USA) supplemented with a protease inhibitor cocktail (539134, Merck Millipore, Germany), following methodologies outlined in previous studies [25]. Protein concentrations were quantitatively assessed through densitometric analysis via ImageJ software. The relative fold differences in expression levels were standardized to the GAPDH expression levels. The primary antibodies utilized included anti-FXR (72105), anti-SHP (3759), anti-SIRT1 (9475), and anti-GAPDH (5174) from Cell Signaling Technology, USA and anti-BSEP (ab155421), anti-MRP2 (ab203397), anti-CYP7A1 (ab65596), and anti-CYP8B1 (ab191910) from Abcam, USA. The secondary antibodies used, specifically the HRP-conjugated AffiniPure goat anti-rabbit or mouse IgG (H + L) (SA00001-2 or SA00001-1), were sourced from Proteintech Group, USA.

### Immunoprecipitation (IP) assay

Liver tissue extraction was conducted via IP lysis buffer (87787, Thermo Fisher Scientific, USA) containing a protease inhibitor cocktail (539134, Merck Millipore, Germany). IP assays utilized antibodies against FXR (72105) and Acetylated-Lysine (9441, Cell Signaling Technology, USA), along with Dynabeads ™ Protein G Immunoprecipitation Kit (10007D, Thermo Fisher Scientific, USA), which strictly followed the protocols provided by the manufacturers.

### Analysis of bile acid pools

The levels of bile acids in the serum, liver, ileum and feces were determined according to a previously established method [26]. The specific methods used were as follows: cold methanol was added to the serum, liver, ileum and feces (tissue samples were first homogenized) for protein precipitation, the mixture was vortexed, the mixture was centrifuged, the entire supernatant was took out, and then vacuum concentration was conducted. Then, a solution of 50% methanol containing the internal standard (CA-d4) was added for dissolution, the mixture was vortexed, and the mixture was centrifuged, after which the supernatant was aspirated for detection. The bile acid concentrations within the samples were quantified via ultra-performance liquid chromatography coupled with triple quadrupole mass spectrometry (AB SCIEX 6500 Series HPLC–MS/MS, USA).

### Luciferase analysis

HEK-293 T cells were maintained at 37 °C in a humidified atmosphere containing 5% CO_2_ and Dulbecco’s Modified Eagle’s Medium (DMEM) enriched with 10% fetal bovine serum (FBS) (Gibco, Carlsbad, California, USA). After 24 h, the cells were transfected with FXR plasmids, RXR-ɑ plasmids, ECRE plasmids or Renilla plasmids in DMEM for 6–8 h. The medium was then replaced with medium containing GW4046 or Cga (10, 25, 50, or 100 μM). Finally, the cell lysates were collected after 24 h and subjected to analysis with a Dual-Lumi™ Luciferase Reporter Gene Assay Kit (RG088S, Beyotime Biotechnology, Shanghai, China) according to the manufacturer’s protocol.

### Statistical analysis

Data analysis was performed via the GraphPad Prism 8 software developed by GraphPad Software, USA. The experimental results are presented as the mean ± SEM. WB results were obtained from 3 independent samples per group, while the results of serum biochemistry, bile acid pool analysis, and qRT-PCR were obtained from six independent samples per group. To evaluate the statistical significance of differences between groups, parametric data were analyzed using either Student’s t test (for two groups) or one-way analysis of variance (ANOVA) (for multiple groups). The *P* < 0.05 was considered statistically significant (*P < 0.05, **P < 0.01, ***P < 0.001) for all statistical tests.

## Results

### Cga attenuates PA-induced liver injury in vivo

**Fig. 1:**
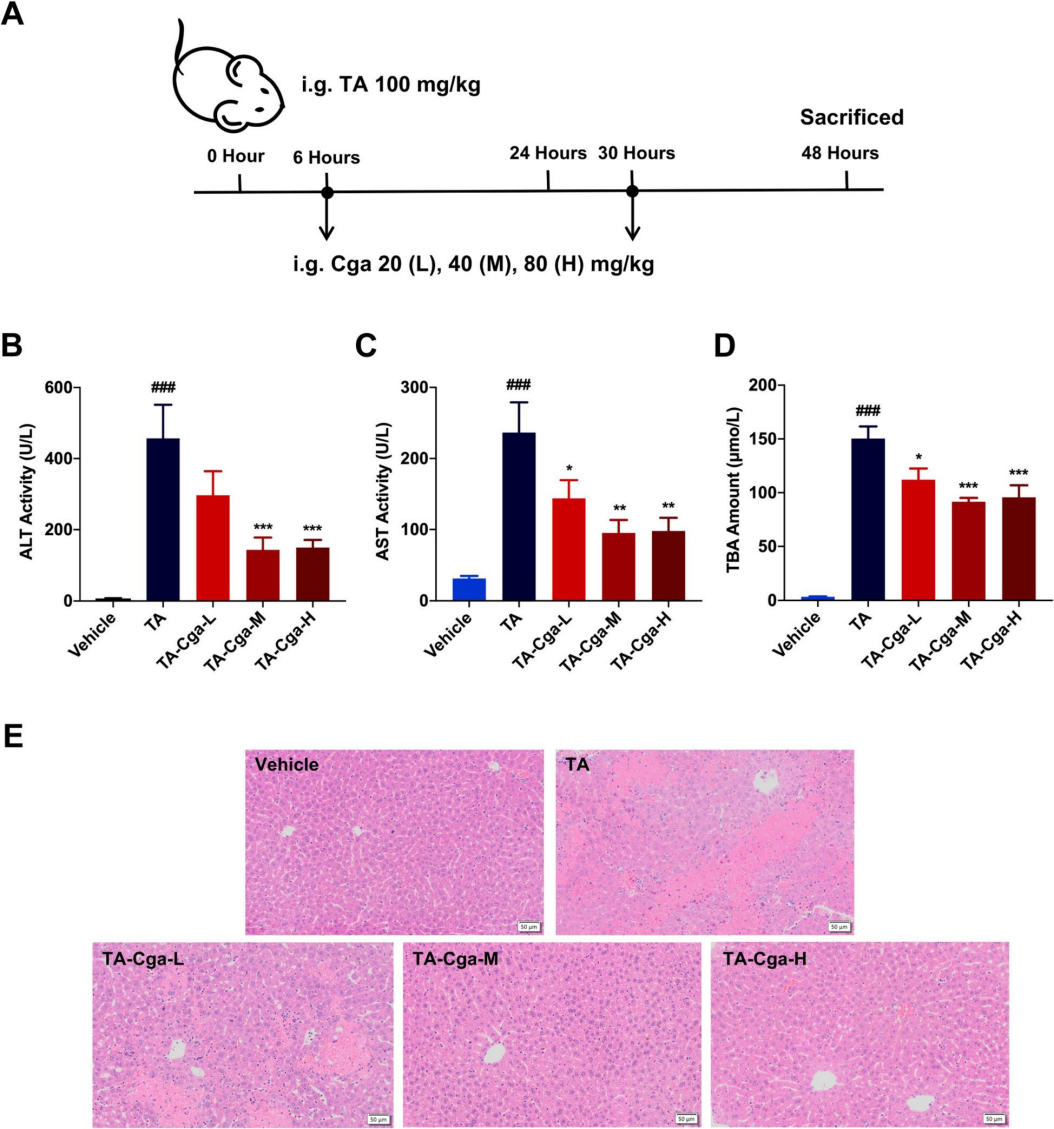
Cga attenuates PA-induced liver injury in vivo*.* **A** The mice were perorally administered TA, and then subjected to two administrations of Cga at 6 h and 30 h post-TA treatment. **B** Serum ALT activity. **C** Serum AST activity. **D** Serum TBA amount. **E** Representative images of H&E-stained liver sections (scale bars, 50 μm). Data are shown as the means ± SEM (n = 6) and analyzed by one-way ANOVA. ^###^*P* < 0.001 vs. Vehicle; *P < 0.05, **P < 0.01, ***P < 0.001 vs. TA

In an experimental study, male C57BL/6J mice were treated with TA at a dose of 100 mg/kg through intragastric administration, followed by the administration of Cga at 20, 40, or 80 mg/kg at 6 and 30 h after the initial TA challenge, as depicted in Fig. 1A. Biochemical analyses of serum revealed a decrease in ALT, AST, and TBA levels in mice that received Cga post-TA exposure (Fig. 1B–D). H&E staining revealed that the PA group displayed intrahepatic hemorrhage, hepatocyte necrosis and detachment of hepatic sinusoidal endothelial cells, compared with the vehicle group. The administration of Cga at 40 mg/kg and 80 mg/kg effectively mitigated these pathological changes, whereas the 20 mg/kg dose resulted in only minor improvements (Fig. 1E, Table 2). These findings affirm the protective role of Cga in ameliorating PA-induced hepatotoxicity in vivo. Given the comparable efficacy of the 40 mg/kg and 80 mg/kg doses, the 40 mg/kg dosage was selected for all further experiments.

**Table 2:**
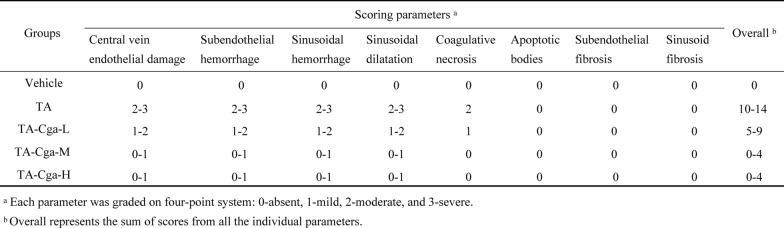
Histological scoring of liver injury in mice

### Cga improves the imbalance of bile acid homeostasis in PA-induced liver injury

**Fig. 2:**
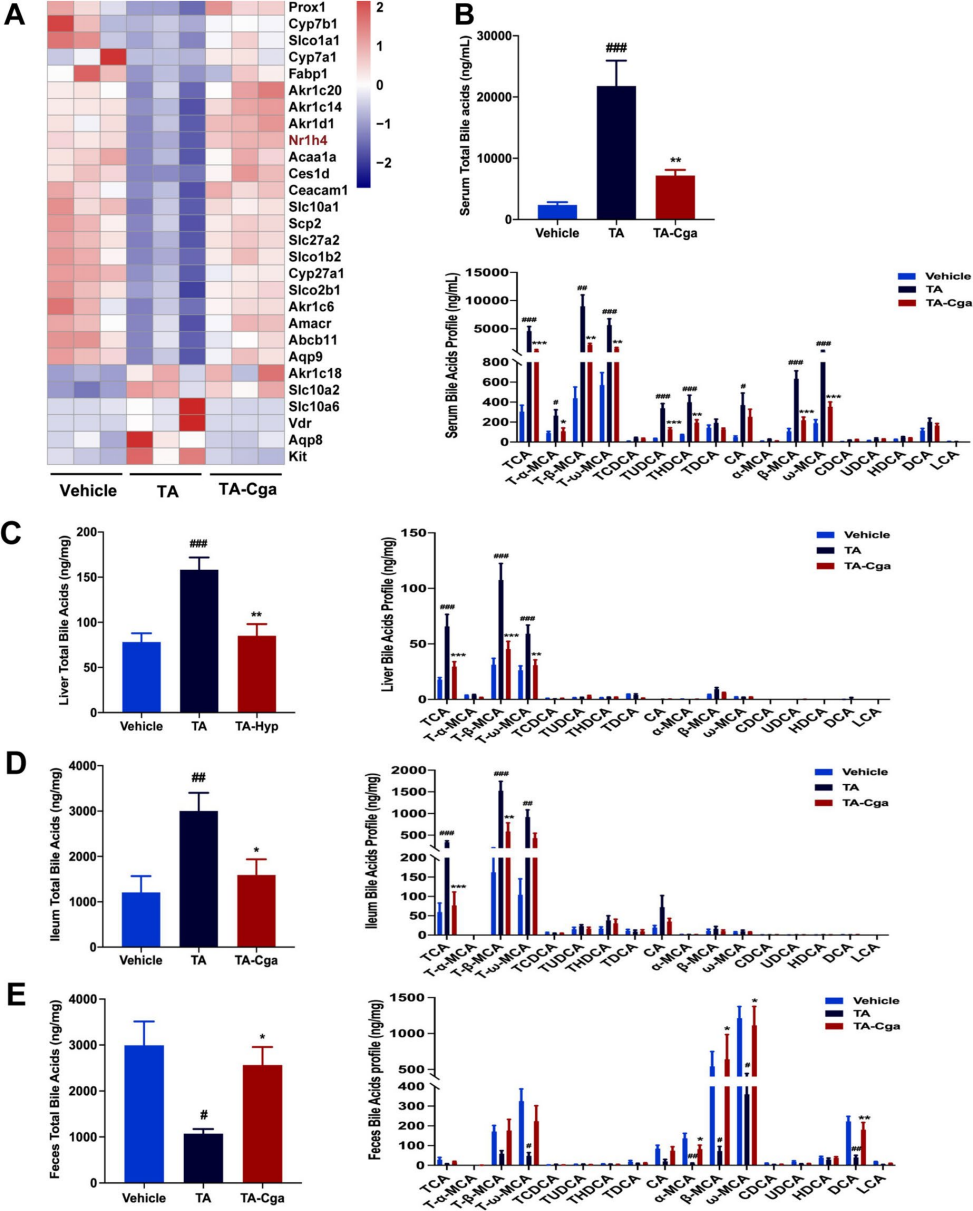
Cga improves the imbalance of bile acid homeostasis in PA-induced liver injury. **A** Heatmap showing the expression profile of genes related to bile acids in different groups. **B** The total bile acids and the profile of bile acids in serum. **C** The total bile acids and the profile of bile acids in liver. **D** The total bile acids and the profile of bile acids in ileum. **E** The total bile acids and the profile of bile acids in feces. Data are shown as the means ± SEM (n = 6) and analyzed by one-way ANOVA. ^**#**^*P* < 0.05, ^**##**^*P* < 0.01, ^**###**^*P* < 0.001 vs. Vehicle; *P < 0.05, **P < 0.01, ***P < 0.001 vs. TA

Research has indicated that disruptions in bile acid balance are a principal contributor to PA-induced acute liver damage [8, 9, 26]. Cga attenuates hepatotoxicity by accelerating the metabolism and efflux of bile acids and decreasing the uptake and synthesis of bile acids [27]. We collected liver tissues from the mice in the vehicle, TA and Cga administration groups for transcriptomic sequencing and analysis of the expression profiles of bile acid-related genes. As shown in Fig. 2A, the expression of bile acid nuclear receptors (e.g., *Nr1h4*), bile acid synthesis and transport genes (e.g., *Cyp7a1*, *Cyp8b1*, *Cyp27a1*, and *Abcb11*), anion transport proteins (e.g., *Slco1a1* and *Slco1b2*), solute carrier family proteins (e.g., *Slc10a1*), and water channel proteins (e.g., *Aqp9*) was downregulated in TA-treated mice, whereas the expression of these bile acid-related genes was upregulated in Cga-treated mice. To confirm that Cga alleviates PA-induced liver injury by regulating bile acid homeostasis, we analyzed the bile acid metabolic profiles of the serum, liver, ileum and feces of the mice. The total bile acid content in the serum, liver and ileum of TA-treated mice was significantly greater than that in vehicle-treated mice, and these increases were reversed by Cga treatment. Specifically, TCA, T-β-MCA, and T-ω-MCA were the bile acids with the most significantly downregulated abundances (Fig. 2B–D). Furthermore, fecal analysis revealed marked alterations in total bile acid levels, with notable increases in partial bile acid levels following Cga administration (Fig. 2E). These findings underscore the role of Cga in reducing bile acid levels in the serum, liver, and ileum, potentially ameliorating metabolic disturbances in mice suffering from PA-induced hepatotoxicity.

### Cga improves the PA-induced imbalance in bile acid homeostasis by activating FXR

**Fig. 3:**
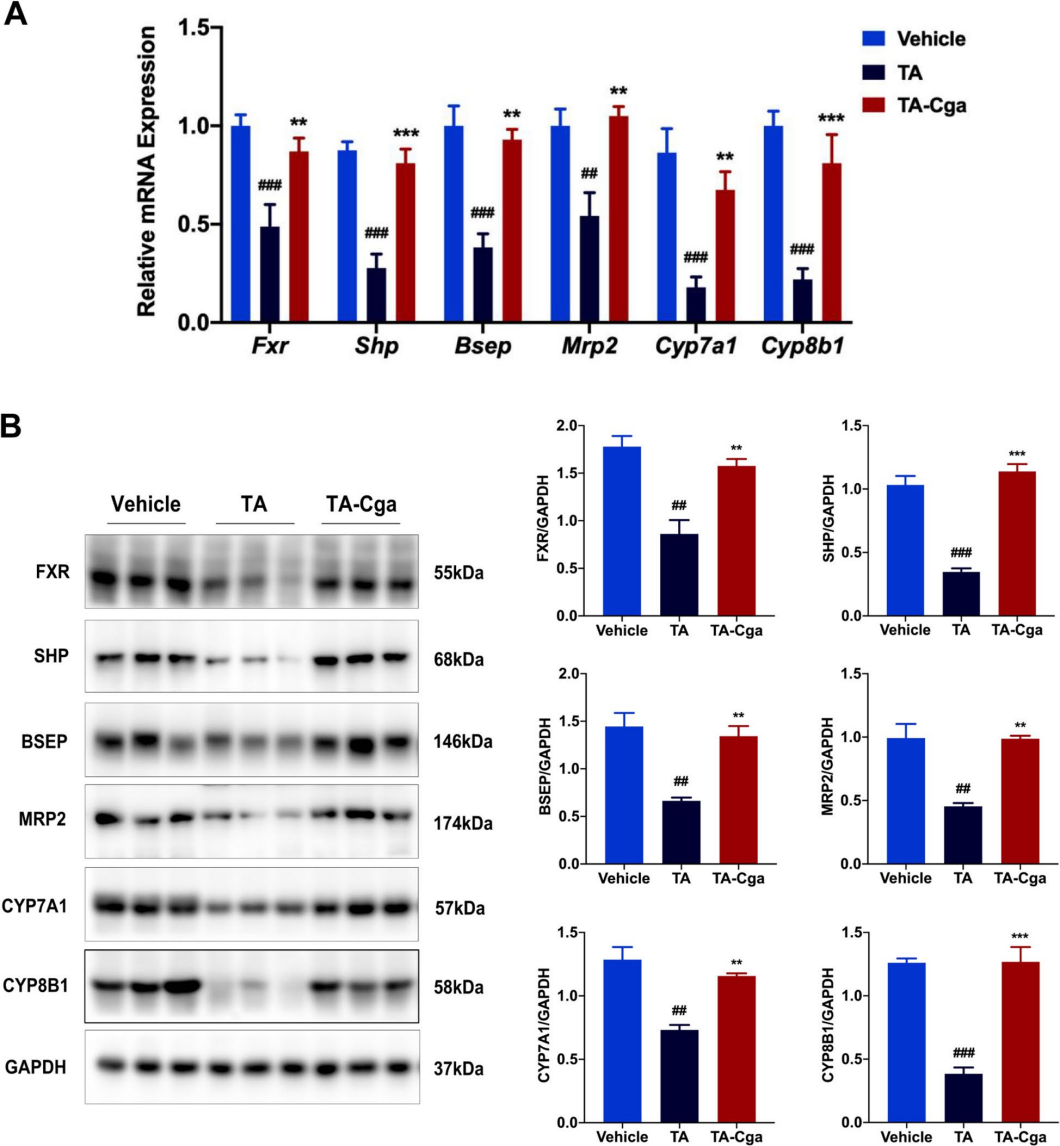
Cga improves the PA-induced imbalance in bile acid homeostasis by activating FXR. **A** The mRNA levels of *Fxr* and its associated genes (*Shp*, *Bsep*, *Mrp2*, *Cyp7a1*, *Cyp8b1*) were evaluated by qRT-PCR (n = 6). **B** The expression of the proteins FXR, SHP, BSEP, MRP2, CYP7A1, CYP8B1 were measured by WB in mouse livers. Bar graphs show summary data (n = 3). Data are shown as the means ± SEM and analyzed by one-way ANOVA. ^**##**^*P* < 0.01, ^**###**^*P* < 0.001 vs. Vehicle; **P < 0.01, ***P < 0.001 vs. TA

On the basis of bile acid-related gene expression profiles (Fig. 2A), we focused on *Nr1h4* (also known as *Fxr*), which regulates bile acid synthesis, transport, reabsorption and other metabolic processes and plays an important role in the maintenance of bile acid homeostasis. We used qRT-PCR and WB assays to investigate whether Cga can mitigate the imbalance in bile acid homeostasis in TA-exposed mice by activating FXR. As shown in Fig. 3A, compared with vehicle treatment, TA treatment significantly decreased the mRNA expression levels of *Fxr* and its downstream target genes (*Shp*, *Bsep*, and *Mrp2*) and bile acid synthesis genes (*Cyp7a1* and *Cyp8b1*). Cga treatment effectively reversed these reductions. Moreover, WB analysis revealed lower FXR, SHP, BSEP, MRP2, CYP7A1, and CYP8B1 protein expression levels in the TA-treated group than in the vehicle-treated group, but these reductions were ameliorated by Cga treatment (Fig. 3B). These results indicate that Cga contributes to the restoration of bile acid homeostasis, which is disrupted by PAs, through the activation of FXR.

### Knocking out *Fxr* blocks the beneficial effects of Cga on PA-induced liver injury

**Fig. 4:**
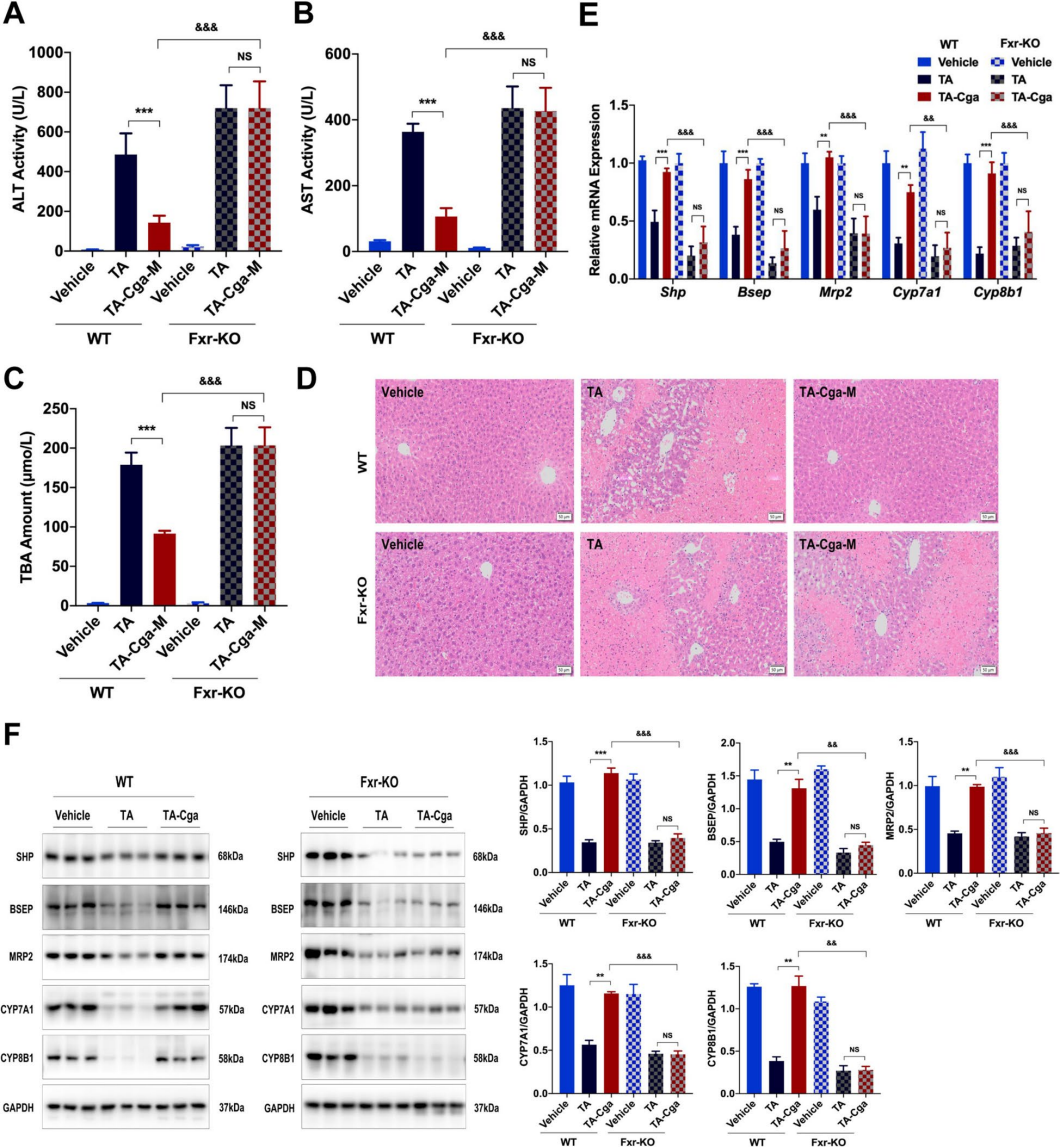
Knocking out *Fxr* blocks the beneficial effects of Cga on PA-induced liver injury. **A** Serum ALT activity. **B** Serum AST activity. **C** Serum TBA amount. **D** Representative images of H&E-stained liver sections (scale bars, 50 μm). **E** The mRNA levels of *Fxr* associated genes (*Shp*, *Bsep*, *Mrp2*, *Cyp7a1*, *Cyp8b1*) were evaluated by qRT-PCR (n = 6). **F** The expression of the proteins SHP, BSEP, MRP2, CYP7A1, CYP8B1 were measured by WB in mouse livers. Bar graphs show summary data (n = 3). Data are shown as the means ± SEM and analyzed by one-way ANOVA. **P < 0.01, ***P < 0.001 vs. TA; ^&&^*P* < 0.01, ^&&&^*P* < 0.001 vs. TA-Hyp

Subsequent investigations focused on determining the influence of FXR activation on the protective effects of Cga against liver damage caused by PAs. Compared with those in WT mice, the increases in the serum ALT, AST, and TBA levels in *Fxr*-KO mice induced by PAs were not mitigated by the administration of Cga (Fig. 4A–C), nor was the degree of hepatocytic necrosis or intrahepatic hemorrhage (Fig. 4D, Table 3). In particular, in *Fxr*-KO mice, the mRNA and protein expression levels of genes related to bile acids were unaffected by Cga treatment (Fig. 4E, F). Fxr was successfully knocked out in all *Fxr*-KO mice, as demonstrated in Supplementary Fig. 1. These results predominantly implicate FXR activation as crucial for the ameliorative impact of Cga in the context of PA-triggered liver damage.

**Table 3:**
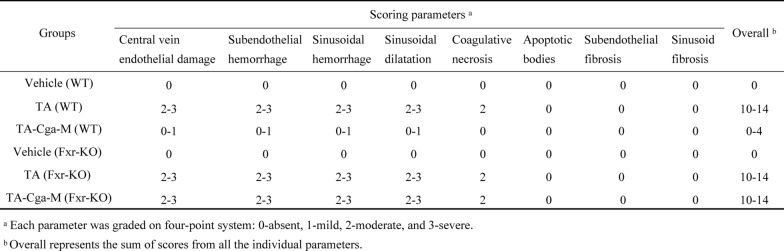
Histological scoring of liver injury in mice

### Cga attenuates PA-induced liver injury by activating the SIRT1/FXR pathway

**Fig. 5:**
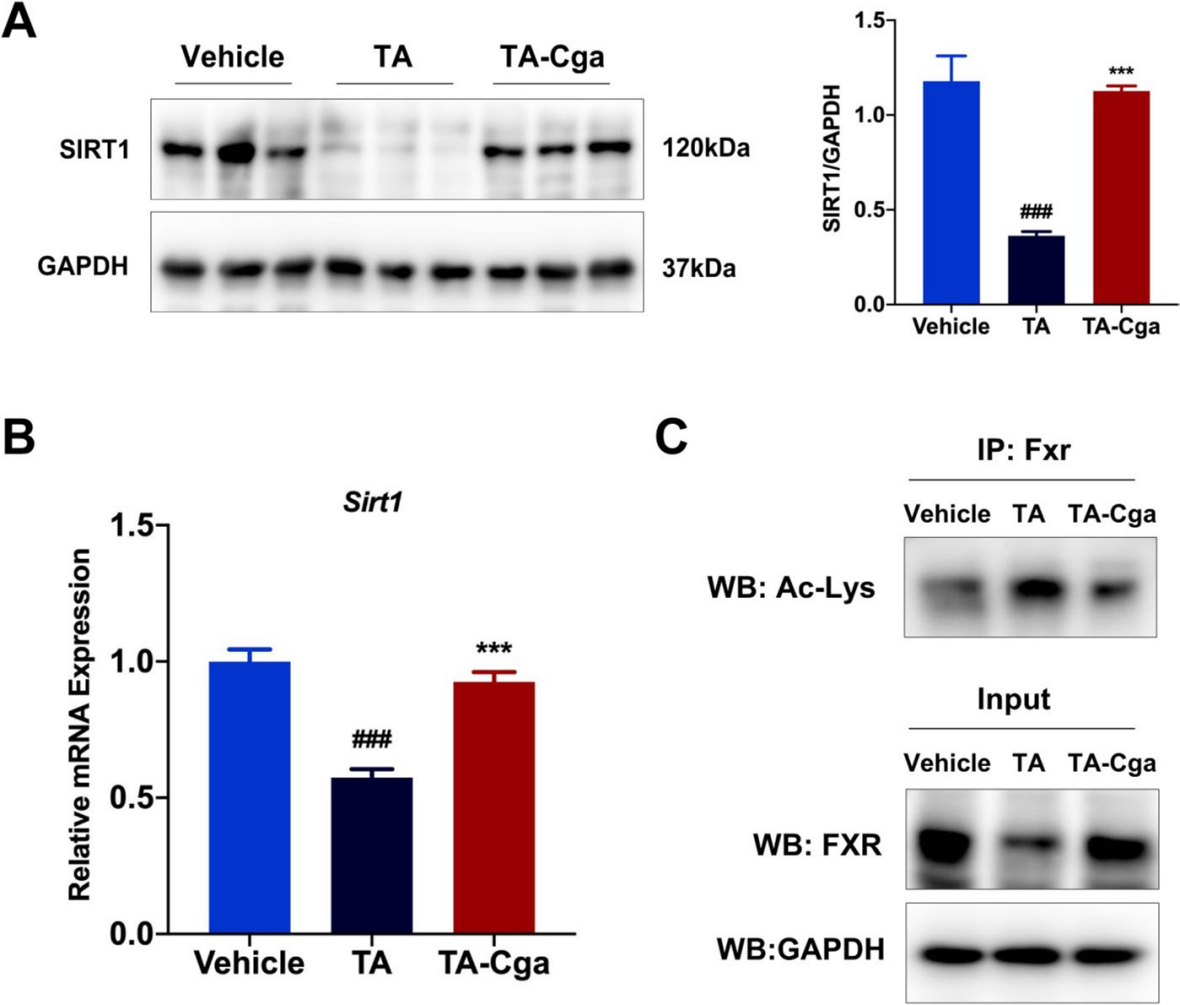
Cga attenuates PA-induced liver injury by activating the SIRT1/FXR pathway. **A** The expression of the protein SIRT1 was measured by WB in mouse livers. Bar graphs show summary data (n = 3). **B** The mRNA level of *Sirt1* was evaluated by qRT-PCR (n = 6). **C** The expression of the proteins Ac-Lys and FXR were measured by IP analysis in mouse livers (n = 3). Data are shown as the means ± SEM and analyzed by one-way ANOVA. ^**###**^*P* < 0.001 vs. Vehicle; ***P < 0.001 vs. TA

Initial investigations revealed the effect of Cga on FXR luciferase reporter plasmid-transfected cells, and the results revealed that Cga was unable to induce FXR activation (Supplementary Fig. 2). The regulation of FXR activity involves a dynamic acetylation/deacetylation process mediated by SIRT1 [14]. SIRT1 is a class III NAD^**+**^-dependent histone deacetylase that tightly regulates bile acid metabolism [28]. As shown in Fig. 5A, PA exposure markedly diminished SIRT1 protein expression in the mouse liver, whereas SIRT1 protein expression was increased in the Cga treatment group. Consistent with the WB results, Cga also reversed the reduction in the mRNA expression of *Sirt1* in PA-induced liver injury (Fig. 5B). Many studies have reported that SIRT1 can modulate FXR-stimulated transcriptional signaling by deacetylating this nuclear receptor and controlling the activation of FXR [14, 16]. IP assays revealed that FXR was hyperacetylated in the PA-treated group compared with the vehicle-treated group, whereas Cga reduced FXR acetylation, indicating that Cga enhances SIRT1-mediated deacetylation of FXR (Fig. 5C). These findings indicate that Cga ameliorates the imbalance in bile acid homeostasis caused by PAs via the SIRT1/FXR signaling pathway.

### Inhibition of SIRT1 expression blocks the beneficial effects of Cga on PA-induced liver injury

**Fig. 6:**
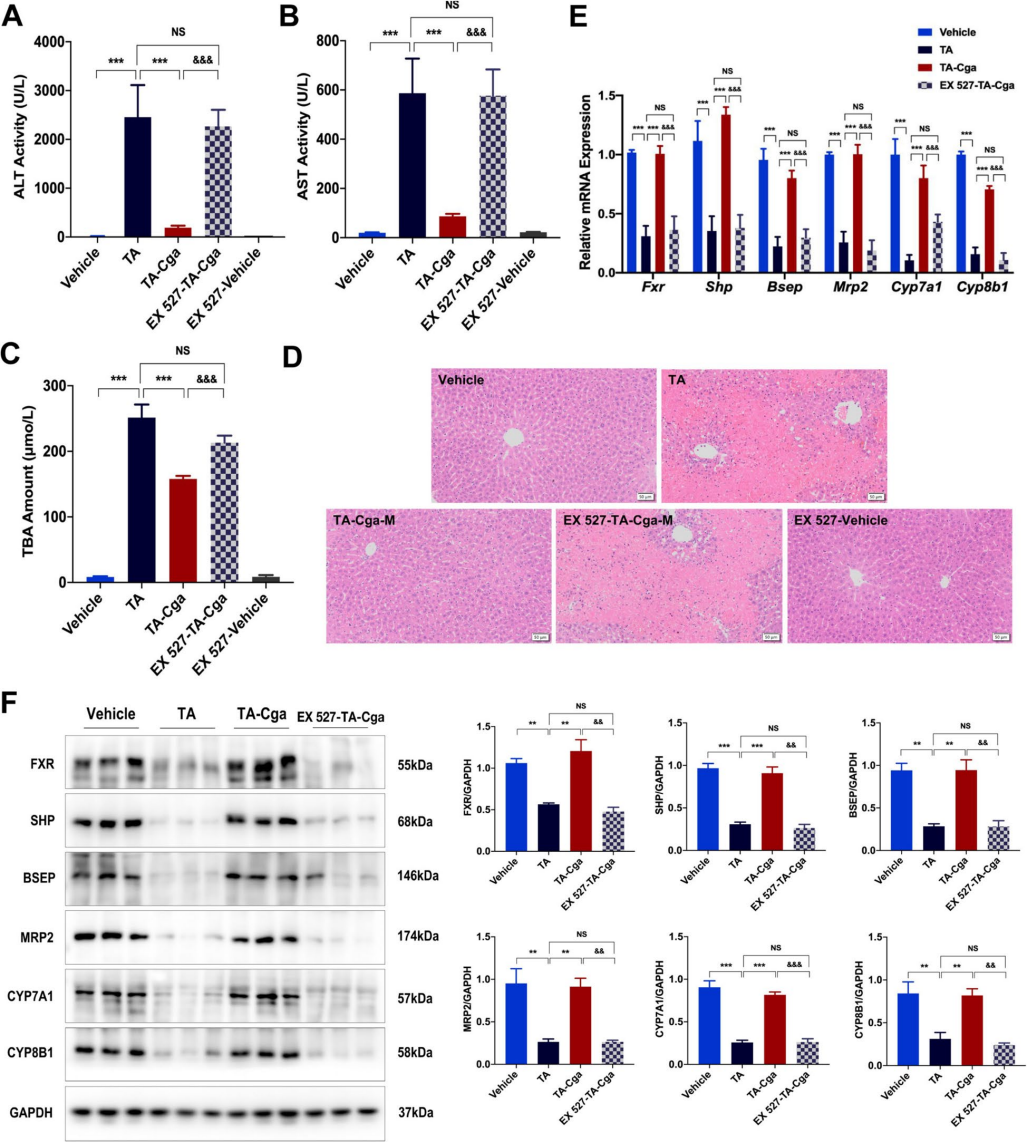
Inhibition of SIRT1 expression blocks the beneficial effects of Cga on PA-induced liver injury. **A** Serum ALT activity. **B** Serum AST activity. **C** Serum TBA amount. **D** Representative images of H&E-stained liver sections (scale bars, 50 μm). **E** The mRNA levels of *Fxr* and its associated genes (*Shp*, *Bsep*, *Mrp2*, *Cyp7a1*, *Cyp8b1*) were evaluated by qRT-PCR (n = 6). **F** The expression of the proteins FXR, SHP, BSEP, MRP2, CYP7A1, CYP8B1 were measured by WB in mouse livers. Bar graphs show summary data (n = 3). Data are shown as the means ± SEM and analyzed by Student's t test or one-way ANOVA. ^**^P < 0.01, ^***^ *P* < 0.001 vs. TA; ^&&^*P* < 0.01, ^&&&^*P* < 0.001 vs. TA-Hyp

To further elucidate the role of SIRT1 in mediating the protective effects of Cga on PA-induced hepatotoxicity, the SIRT1 inhibitor EX 527 was administered to mice to suppress SIRT1 activity. This intervention abrogated the hepatoprotective benefits of Cga, as evidenced by the results of serum biochemical assays and H&E staining (Fig. 6A–D, Table 4). Furthermore, EX 527 reversed the increases in the mRNA and protein expression levels of FXR, SHP, BSEP, MRP2, CYP7A1, and CYP8B1 induced by Cga in the context of PA-related liver injury (Fig. 6E, F). Collectively, these results confirm that blocking SIRT1 activity diminishes the protective effects of Cga against PA-induced hepatotoxicity, indicating that the SIRT1/FXR pathway predominantly facilitates the beneficial effects of Cga on PA-triggered hepatotoxicity.

**Table 4:**
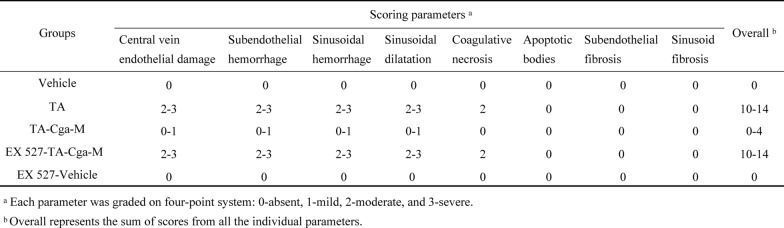
Histological scoring of liver injury in mice

## Discussion

Our research revealed that Cga can attenuate the PA-induced imbalance in bile acid homeostasis through FXR activation. This is due to an increase in SIRT1-mediated FXR deacetylation, which alleviates the hepatotoxicity of PAs in mice. These findings suggest that Cga represents a viable new treatment option for liver damage caused by PAs and that focusing on the FXR/SIRT1 pathway could offer an effective approach for managing liver injuries resulting from medications.

Maintaining equilibrium in bile acid homeostasis is crucial for the proper functioning of a healthy liver and involves enzymes that are involved in the production and detoxification of bile acids, as well as sinusoidal and canalicular transporters that mediate bile acid flow. Recent studies have increasingly associated disruptions in bile acid homeostasis with hepatotoxicity induced by PAs [29–31]. PAs have been found to disrupt bile acid homeostasis and secretion by downregulating the expression of hepatobiliary transporter enzymes that are essential for bile acid synthesis and conjugation and transcription regulators [30]. Thus, PAs have demonstrated a significant capacity to alter the expression of genes associated with cholestasis and to reduce bile acid levels [31]. Moreover, our group and others proposed that total bile acids could serve as a sensitive index of liver functionality upon PA exposure [32, 33]. The synthesis of bile acids begins with the CYP7A1-mediated formation of 7α-hydroxycholesterol, which subsequently leads to the production of two primary bile acids, cholic acid and chenodeoxycholic acid [34]. The biosynthesis of cholic acid involves hydroxylation at the 12th position of the steroid ring, which is catalyzed by CYP8B1 [35]. Chenodeoxycholic acid facilitates the interaction between the ligand-binding domain of FXR and steroid receptor coactivator-1 with other nuclear receptors [36, 37]. Upon binding to bile acids, FXR suppresses the transcription of CYP7A1 and CYP8B1, but promotes the transcription of the hepatic efflux transporters BSEP and MRP2, thus preventing the accumulation of cytotoxic bile acids within hepatocytes under normal conditions [38]. Accordingly, our research confirms that an imbalance in bile acid homeostasis occurs in PA-treated mice, suggesting that interventions aimed at bile acid regulation could serve as potent treatments for PA-related liver damage.

Cga is a bioactive phenolic acid that has been extensive applied in medical and pharmaceutical fields, and its efficacy in treating nonalcoholic steatohepatitis and cholestasis has been well documented. Studies have shown that Cga reverses nonalcoholic steatohepatitis by regulating bile acid signaling [39]; additionally, Cga attenuates cholestasis by decreasing bile acid uptake and synthesis while enhancing the bile acid metabolism and efflux [27]. Therefore, by examining how Cga improved PA-induced hepatotoxicity, we combined the results of the expression profiles of bile acid-related genes and bile acid metabolomic analyses. These results demonstrate that Cga attenuates PA-induced liver damage through the regulation of bile acid homeostasis, indicating that Cga can inhibit the intrahepatic synthesis of bile acids and reduce ileal reabsorption. The primary regulator of bile acid homeostasis, FXR, which mediates an adaptive response aimed at reducing the bile acid pool size by inhibiting bile acid synthesis and modulating its transport [40]. A recent study revealed that senecionine, a principal PA, is highly potent in binding with FXR. Exposure to senecionine modified the hepatic mRNA expression levels of *Fxr* downstream genes involved in bile acid biosynthesis (*Shp*, *Cyp7a1* and *Cyp8a1*) and bile acid secretion (*Mrp2* and *Bsep*). In addition, a significant reduction in hepatic FXR expression was observed in patients with PA-HSOS [32]. These findings suggest the potential of FXR activation as a novel therapeutic approach for PA-related liver damage. We investigated whether Cga ameliorates PA-induced liver injury by activating FXR. First, Cga increased FXR mRNA and protein expression levels in PA-treated mice. Second, Cga increased the expression of bile acid metabolism-related genes downstream of FXR. Third, the Cga-mediated protection against PA-induced liver injury was completely diminished in *Fxr* knockout mice. These outcomes align with prior reports that link FXR deficiency with alterations in bile acid pool composition and increased hepatic toxicity [41, 42]. Taken together, these results confirm the critical role of FXR in attenuating PA-induced hepatotoxicity and that Cga-induced FXR activation contributes to its protection against PA-induced liver injury.

Recent studies highlight the critical role of FXR agonists in treating a variety of liver-related conditions, including primary sclerosing cholangitis, primary biliary cirrhosis, cholestatic liver disorders, and nonalcoholic fatty liver disease, and preventing gallstone formation. Our results have shown that Cga is unable to activate FXR. FXR regulation is subject to a dynamic deacetylation mechanism coordinated by SIRT1, which is crucial for FXR-DNA binding and the transcription of target genes; this mechanism also governs the proteasomal degradation of FXR [14]. It has been reported that SIRT1 activation alleviates cholestatic liver injury, which is associated with bile acid homeostasis, including enhancing FXR signaling, increasing the hydrophilicity of hepatic bile acid and decreasing the plasma bile acid concentration via increased bile acid excretion into the urine [43]. Thus, SIRT1 activation may be a novel therapeutic target for PA-induced liver injury.

Exploration of the mechanism by which Cga mitigates PA-induced hepatic damage via bile acid homeostasis included an examination of its impact on SIRT1 expression levels. These results suggest that Cga increases the liver SIRT1 content in PA-induced hepatotoxicity. A previous study showed that SIRT1-mediated deacetylation of FXR is decreased in cholestatic livers [43]. Our results revealed that FXR was hyperacetylated in the livers of PA-treated mice and that it reduced the recruitment of SIRT1 to FXR; moreover, Cga treatment effectively reversed these reductions. These results indicate that Cga enhances the SIRT1-mediated deacetylation of FXR, which may contribute to its ability to alleviate PA-induced liver injury.

To confirm whether SIRT1 is crucial in PA-induced hepatic damage, a SIRT1 inhibitor, EX 527, was used. Investigations involving serum levels of ALT, AST, and TBA, alongside histological assessments of liver tissue, revealed that EX 527 partially abrogated the beneficial effect of Cga on PA-induced hepatotoxicity. Furthermore, Cga-induced activation of the FXR signaling pathway was also inhibited by EX 527. These results imply that the Cga-activated SIRT1-mediated FXR signaling pathway plays an important role in the development of liver damage induced by PAs.

This study has limitations. It remains unclear whether Cga directly activates SIRT1 in the context of PA-induced liver injury. While Cga has been shown to potentially activate SIRT1 by forming complexes, thereby protecting primary neurons from oxygen–glucose deprivation damage [44], further studies are essential to elucidate how Cga modulates SIRT1 to mitigate the hepatotoxicity caused by PA. As the findings presented here were derived from mouse-based studies, the suggested mechanisms should be validated in PA-HSOS patients to confirm whether SIRT1 could serve as a viable clinical target for treating PA-induced liver damage.

## Conclusion

In summary, the results of this study show that Cga ameliorated liver injury induced by PAs through the activation of the SIRT1/FXR signaling pathway. Our results support the potential of Cga as an effective treatment option for liver injuries caused by PAs in clinical settings.

## Supplementary Information

Supplementary Material 1: Fig. 1. *Fxr* was knocked out in all *Fxr*-KO mice. The expression of the protein FXR was measured by WB in mouse livers (n = 6).

Supplementary Material 2: Fig. 2. The effect of Cga on transfected FXR plasmid cells (n = 3). Data are shown as the means ± SD and analyzed by Student's t-test. ^### ^*P* < 0.001 vs. Vehicle.

## Data Availability

The research data used to support the findings are available from the corresponding author upon request.

## Abbreviations

ASBT: Apical sodium-dependent bile acid transporter
ALT: Alanine aminotransferase
AST: Aspartate aminotransferase
BSEP: Bile salt export pump
Cga: Chlorogenic acid
DMEM: Dulbecco’s Modified Eagle’s Medium
FXR: Farnesoid X receptor
Fxr-KO: Whole-body Fxr knockout
FBS: Fetal bovine serum
HSOS: Hepatic sinusoidal obstruction syndrome
H&E: Hematoxylin–eosin
IP: Immunoprecipitation
MRP2: Multidrug resistance-associated protein 2
OSTα/β: Organic solute transporter α and β heterodimer
PAs: Pyrrolizidine alkaloids
qRT-PCR: Quantitative real-time PCR
SIRT1: Sirtuin 1
SHP: Short heterodimer partner
SPF: Specific pathogen-free
TA: Total alkaloid extract
TBA: Total bile acid
WT: Wild-type
WB: Western blotting
