# Supplementary figures and images for: Chlorogenic acid attenuates pyrrolizidine alkaloid-induced liver injury through modulation of the SIRT1/FXR signaling pathway
Source: Chin Med. 2025 Mar 12;20:34. doi: 10.1186/s13020-025-01077-2 (PMC11899315; doi:10.1186/s13020-025-01077-2)

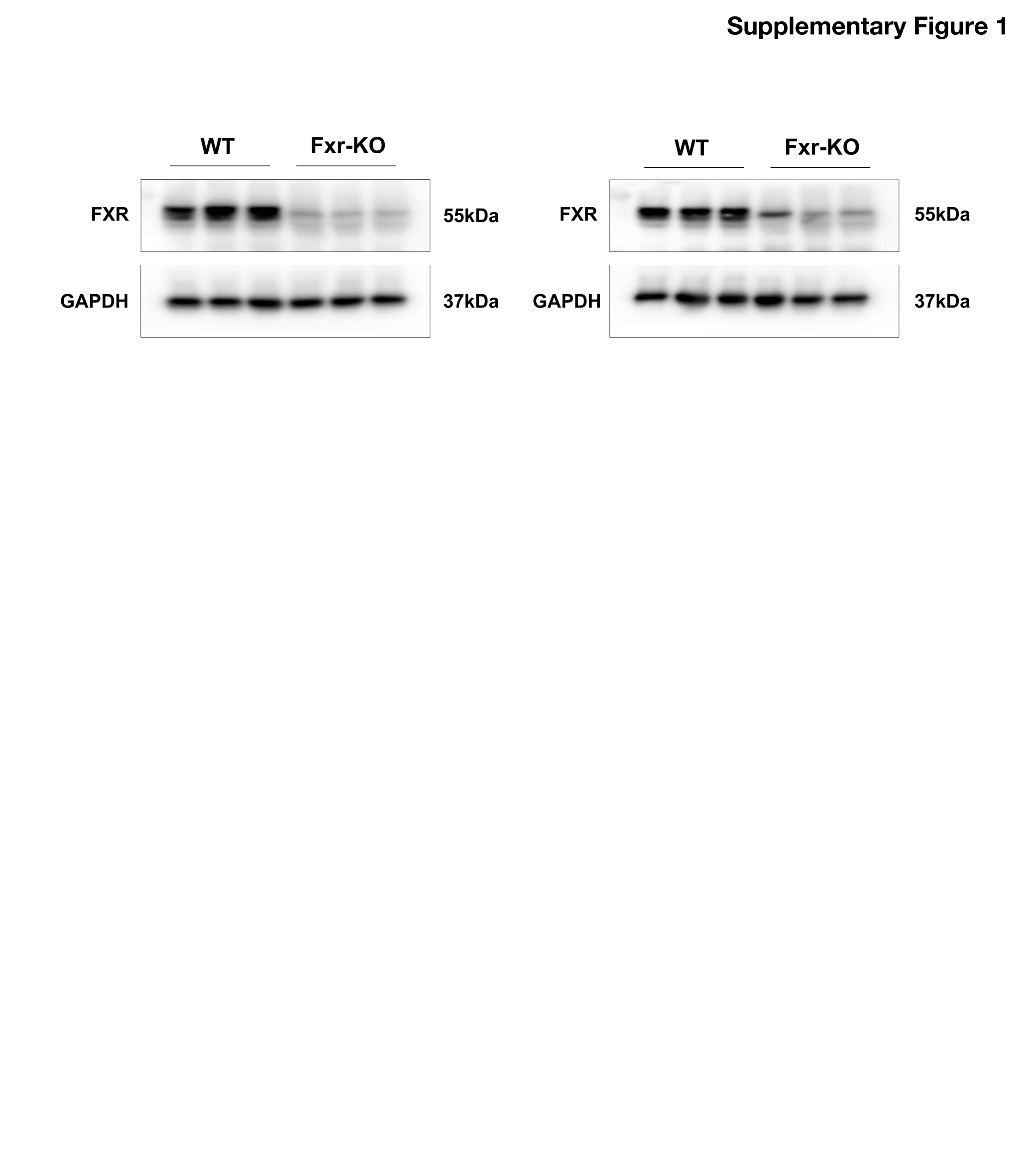

Supplement: Supplementary file 1 — Supplementary Material 1: Fig. 1. Fxr was knocked out in all Fxr-KO mice. The expression of the protein FXR was measured by WB in mouse livers (n = 6). [file 13020_2025_1077_MOESM1_ESM.tif]

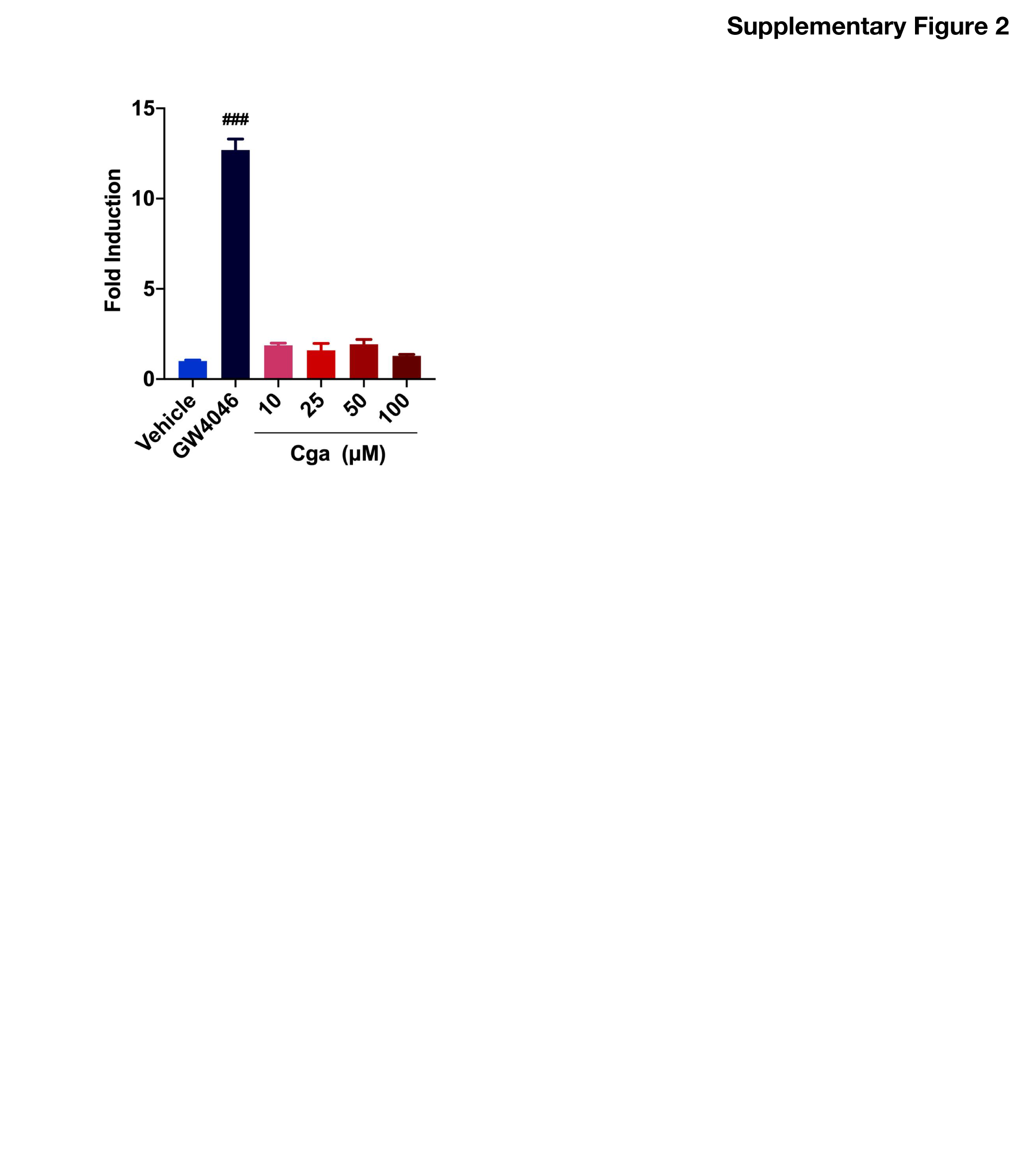

Supplement: Supplementary file 2 — Supplementary Material 2: Fig. 2. The effect of Cga on transfected FXR plasmid cells (n = 3). Data are shown as the means ± SD and analyzed by Student's t-test. ### P < 0.001 vs. Vehicle. [file 13020_2025_1077_MOESM2_ESM.tif]
